# High Annual Risk of Tuberculosis Infection among Nursing Students in South India: A Cohort Study

**DOI:** 10.1371/journal.pone.0026199

**Published:** 2011-10-12

**Authors:** Devasahayam J. Christopher, Prince James, Peter Daley, Lois Armstrong, Barney T. J. Isaac, Balamugesh Thangakunam, Beulah Premkumar, Alice Zwerling, Madhukar Pai

**Affiliations:** 1 Christian Medical College, Vellore, India; 2 Memorial University, St. John's, New Foundland, Canada; 3 McGill University, Montreal, Quebec, Canada; National Institute for Infectious Diseases (L. Spallanzani), Italy

## Abstract

**Background:**

Nurses in developing countries are frequently exposed to infectious tuberculosis (TB) patients, and have a high prevalence of TB infection. To estimate the incidence of *new* TB infection, we recruited a cohort of young nursing trainees at the Christian Medical College in Southern India. Annual tuberculin skin testing (TST) was conducted to assess the annual risk of TB infection (ARTI) in this cohort.

**Methodology/Principal Findings:**

436 nursing students completed baseline two-step TST testing in 2007 and 217 were TST-negative and therefore eligible for repeat testing in 2008. 181 subjects completed a detailed questionnaire on exposure to tuberculosis from workplace and social contacts. A physician verified the questionnaire and clinical log book and screened the subjects for symptoms of active TB. The majority of nursing students (96.7%) were females, almost 84% were under 22 years of age, and 80% had BCG scars. Among those students who underwent repeat testing in 2008, 14 had TST conversions using the ATS/CDC/IDSA conversion definition of 10 mm or greater increase over baseline. The ARTI was therefore estimated as 7.8% (95%CI: 4.3–12.8%). This was significantly higher than the national average ARTI of 1.5%. Sputum collection and caring for pulmonary TB patients were both high risk activities that were associated with TST conversions in this young nursing cohort.

**Conclusions:**

Our study showed a high ARTI among young nursing trainees, substantially higher than that seen in the general Indian population. Indian healthcare providers and the Indian Revised National TB Control Programme will need to implement internationally recommended TB infection control interventions to protect its health care workforce.

## Introduction

Tuberculosis (TB) continues to be a global killer, with over 9 million new cases of active disease diagnosed every year.[1] Health care workers (HCW) serve on the front lines of the battle against TB, and the risk of infection among HCWs is especially high. [2], [3] In a systematic review of studies from low and middle income countries, the annual risk of TB infection (LTBI) was found to vary between 0.5% to 14.3% in HCWs, while the annual incidence of TB disease ranged from 69–5,780 cases per 100 000 HCWs.[2] In recent years, the emergence of multi drug-resistant (MDR) and extensively drug resistant (XDR) TB strains have sparked a renewed interest in and emphasis on the protection of the healthcare workforce. In 2009, the World Health Organization released guidelines on TB Infection Control (TBIC) in resource limited settings, and is now actively promoting TBIC programmes.[4] TB infection control is also included in the 3-Is initiative (infection control, isoniazid preventive therapy [IPT], and intensive case finding) by the WHO, especially in countries with high prevalence of HIV.

HCWs in high TB burden settings are at higher risk of developing LTBI, compared with the general population, because of their exposure to large numbers of smear-positive TB cases managed at hospitals or health care centers. Nurses, in particular, spend a lot of time caring for smear positive and smear negative pulmonary TB patients, and are particularly at risk for acquisition of LTBI[2]. A study from central India reported LTBI prevalence proportions of around 40% among Indian HCWs using either the tuberculin skin test (TST) or the novel interferon-gamma release assays (IGRA)[5]. The study found an increased prevalence of LTBI with increasing age, and years worked in health care. [5] Similarly, the annual risk of TB infection (ARTI) among medical and nursing trainees was found to be 5%, 3 times higher than the 1.5% estimated ARTI for the general population in India. [6]

Traditionally, screening HCWs for LTBI was done with the Tuberculin Skin Test (TST), however there are some limitations for its use in serial testing HCWs, including: complications with boosting and conversions.[7] In recent years, the introduction of novel IGRAs have provided an alternative to the 100 year old TST. There are two IGRAs commercially available: the TSPOT.TB assay (Oxford Immunotech, Abingdon, UK) and the QuantiFERON-TB Gold In-Tube (QFT) assay (Cellestis Ltd, Carnegie, Australia). These IGRA assays have some advantages over the TST including not being affected by prior BCG vaccination or non TB mycobacterial (NTM) infection. IGRAs are in vitro tests and eliminate concerns regarding adverse events or boosting and do not require a return visit.[8], [9], [10], [11] The use of IGRAs in HCWs is increasing and there are several published studies and two systematic reviews published.[12], [13] While these assays show promise for screening in low incidence settings, they appear to have lower sensitivity in high incidence settings such as India[8], [12] and predictive (prognostic) value of both TST and IGRA for identifying those at highest risk of progressing to active TB disease appear to be limited, especially in high TB burden settings.[14]

In an effort to better understand nosocomial TB among nursing trainees in Southern India, we initiated a cohort study among nursing students training at a large tertiary care hospital. Our earlier publication on this cohort of young nursing trainees reported a high LTBI prevalence of 47.8% using latent class analysis (95% credible interval: 17.8%–65.6%).[15] We now report the results from annual testing using TST in this cohort of nursing trainees.[15]

## Methods

### Study Design and Ethics Approvals

Our study was conducted at the Christian Medical College (CMC) in Vellore, India. Nursing students at the college were approached for enrollment in the study in the year 2007 and were followed up prospectively at yearly intervals. TST was repeated annually, but only if previously negative. Students with a positive TST result were assessed for active TB, and TST was not repeated in these individuals. Baseline demographics and results of cross-sectional testing of this cohort have been published previously.[15]

All study participants were aged 18 or more, and all participants provided written informed consent. The study protocol was approved by the institutional review boards at both Christian Medical College Hospital, Vellore, India and McGill University, Montreal, Canada. All clinical investigations were conducted according to the principles expressed in the Declaration of Helsinki.

#### Setting & Study Participants

The CMC is a large (2200 beds) tertiary referral medical school at Vellore, a town located in the Tamil Nadu state of Southern India. The district of Vellore has an annual TB case detection rate of 148/100,000.[16] However the hospital's patient population comes from all over India with many patients travelling substantial distances. In a previous report, we estimated a baseline prevalence of 47.8–50.2% TST positivity (95%CI: 17.8 to 65.6%), for this cohort.[15] An earlier report from the CMC described 126 cases of active TB among various health care workers at the institution over a ten year period.[17] Hospital infection control policy states that smear positive pulmonary TB patients should be admitted to the isolation ward or a single room, with closed doors and open windows, and staff are instructed to wear surgical masks when entering the room. However due to space and resource constraints, the policy is not universally implemented.

Student nurses from all 6 programs offered at the College of Nursing, CMC, Vellore were approached for inclusion in the study, including: Nursing diploma, BSc, Post diploma BSc courses, fellowship courses, MSc, and Doctoral (PhD) programs. Students with a past history of TB were excluded from the study at recruitment. The remaining eligible nursing students signed written consent forms to participate in the prospective cohort.

### Measurement of exposure to TB

All nursing students at CMC routinely maintain detailed clinical log books, which were used to collect information regarding the students' likelihood of TB exposure. Students recorded if they had had direct contact with a smear positive TB patient, and the number of days spent working on various medical wards.

Data were ascertained from clinical log books at baseline and at testing 1 year later. Log books helped identify students who had been exposed to TB and to quantify the number of days spent caring for pulmonary TB patients, since baseline testing. Other exposure information ascertained included: days worked on isolation, pulmonary medicine, and general medicine wards, number of times performed or assisted in sputum collection, all since baseline testing. At both baseline and annual testing, students were asked about exposure to TB outside the hospital setting.

### TST testing & Interpretation

Students were tested with TST at baseline using the two step TST protocol.[18] Two Tuberculin Units (0.1 ml) of RT23 PPD (Staten Serum Institute, Copenhagen) was injected intra-dermally. After 48–72 hours the induration was measured by a trained reader. An induration of ≥10 mm was considered positive at baseline. If TST was negative (<10 mm) at baseline, TST was then repeated at approximately 1 year post baseline. TST was not performed at 1 year if students had been TST positive at baseline. TST conversion was defined as a baseline TST induration of <10 mm, and follow-up TST of ≥10 mm, with an increase of 10 mm, as per ATS/CDC/IDSA standards.[19]

### IGRA testing (QFT)

Students were tested with the QuantiFERON-TB Gold In-Tube (QFT) test (Cellestis Ltd, Carnegie, Australia). QFT was performed per manufacturer's instructions, and the QFT was considered positive if the Interferon-gamma response of TB antigen minus the nil was ≥0.35 IU/mL. QFT was performed on all recruits at the annual screening in 2008. Given only one time point with QFT results we were not able to look at QFT conversions/reversions in this cohort, but such analyses will be possible with subsequent follow-up of this same cohort.

### Management of TST Conversions

Isoniazid (INH) preventive therapy is not routinely offered to HCWs with LTBI in India because of the high background prevalence of LTBI, risks associated with drug toxicity, concern about wide-spread resistance to INH, chances of poor compliance, a high likelihood for re-exposure and re-infection, and lack of evidence that preventive therapy has long term efficacy in TB endemic populations. However, recent conversions are associated with an elevated risk of developing active TB. Therefore, students who had TST conversions upon testing at 1 year were assessed to rule out active TB and then offered preventive therapy using 4 months of Rifampicin, one of the acceptable preventive therapy regimens.[20] All students on preventive therapy were closely followed for adverse events by clinicians in the department of pulmonary medicine.

### Statistical analysis

Clinical and demographic data, along with variables concerning exposure to TB for the cohort were summarized using descriptive statistics. The frequency distribution of TST measurements were displayed graphically as histograms. The main outcome was annual risk of TB infection (ARTI), with 95% confidence intervals, estimated using TST conversions as defined above. This was followed by an analysis to assess the major risk factors for TST conversions. First, univariate regression was used to assess a potential relationship between TB exposure and known clinical and occupational risk variables and a TST conversion. Finally a multivariate logistic model was fit to assess the relationship between TST conversions and known occupational risk factors for TB, variables such as age and BCG vaccination were included due to their a priori association with TST positivity despite not reaching statistical significance in univariate models. All analyses were performed using Stata 11 (Stata Corp, Texas, USA).

## Results

### Description of the cohort

**Figure 1:**
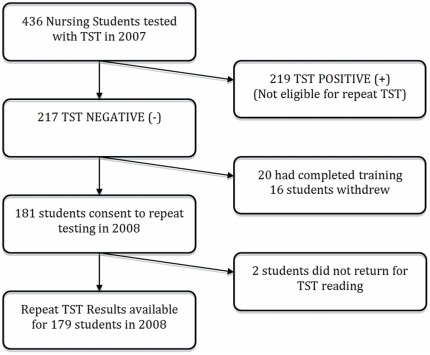
Flow chart of study participants.

Nursing students who were TST negative after baseline two-step testing in 2007 were approached for repeat testing in 2008.[15] Figure 1 shows the flow of participants included in the analysis. Two hundred and sixteen nursing students were TST negative and eligible for repeat testing with the TST in 2008. Nineteen nursing students had completed their training and were no longer working at the institution. A further 16 students withdrew from the study, while the remaining 181 nursing students consented to repeat testing, and 179 completed repeat testing with the TST in 2008.

The cohort was predominantly female (96.7%), 83.8% were under 22 years of age, and 80% of students had evidence of BCG vaccination. All study participants were aged 18 or more. The majority of students were enrolled in the diploma or BSc nursing programs, and the mean time spent working in health care was 34.5 months (Range:13–193; Median  = 28 IQR = 17–39). Tables 1 and 2 provide complete information on participant characteristics at time of repeat testing in 2008.

**Table 1:** Characteristics of study participants who underwent repeat tuberculin testing in 2008.

Variable	N = 179	%
**Sex**		
Female	173	96.65
Male	6	3.35
**Age (yrs)**		
18	26	14.53
19	46	25.7
20	44	24.58
21	34	18.99
22 and older	29	16.20
**BMI (kg/m^2^)**		
< = 19	53	29.61
>19	126	70.39
**Education (highest level completed)**		
Class 12	162	90.5
Diploma	12	6.7
Bachelor degree	4	2.23
Masters degree	1	0.56
**Current Nursing Course**		
Diploma	89	49.72
BSc	77	43.02
Post Diploma BSc	9	5.03
Post diploma specialty	1	0.56
MSc	3	1.68
**Average household monthly income (Indian Rupees)**		
<5000	64	35.75
5000–10,000	62	34.64
10,000–20,000	28	15.64
20,000–30,000	12	6.7
>30,000	9	5.03
Refused	4	2.23

**Table 2:** Prevalence of risk factors & TB exposure at repeat tuberculin testing in 2008.

Variable	N = 179	%
BCG vaccination		
No	8	4.5
At birth	140	78.2
After birth	3	1.7
Unknown	28	15.6
Total months in health care	Median = 28IQR: 17–39	
Direct contact with smear positive TB pt since last testing		
No	54	30.2
Yes	65	36.3
Yes, but unsure if smear positive	32	17.9
Don't Know	28	15.6
Days spent caring for pulmonary TB since last testing	Median = 4IQR: 0–9	
Performed or assisted with sputum collection since last testing		
Never	101	56.4
<10 times	75	41.9
>10 times	3	1.67
Days spent working on isolation wards since last testing	Median = 4IQR: 0–6	
Days spent working on pulmonary medicine wards since last testing	Median = 0IQR = 0 (Range:0–14)	
Reported contact with TB outside the hospital setting	5	2.8

### TB Exposure and Risk Factors

Nursing students maintained detailed clinical log books, which were used to ascertain days spent working on different wards and exposure to smear positive pulmonary patients. Sixty-five students (36.3%) had had direct contact with a smear positive TB patient as part of their nurse training since baseline testing in 2007. A further 32 students (17.9%) had had direct contact with TB patients, but were unsure if they were smear positive. Students reported a mean of 6.04 days caring for pulmonary TB patients since baseline testing (Range: 0–60 days; Median  = 4 IQR = 0–9). Since baseline testing, students reported working a mean of 4.45 days on isolation wards (Range: 0–28 days; Median  = 4 IQR = 0–6) and a mean of 0.76 days working in pulmonary medicine wards (Range: 0–14 days; Median  =  0 IQR =  0). Almost half of the cohort (43.57%) had performed or assisted with sputum collection since last TST testing. Only five students (2.79%) recalled contact with TB outside the hospital setting since baseline testing, presumably because they were all residing in the institutional hostels (dormitories).

### TST Conversions and Annual Risk of TB Infection (ARTI)

**Figure 2:**
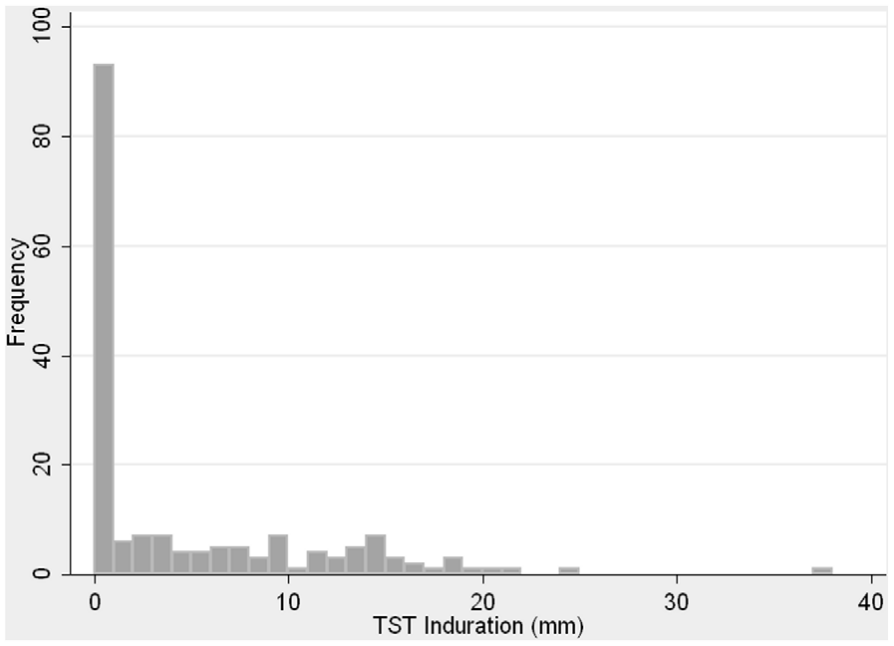
Frequency distribution of tuberculin skin test reactions at annual testing in 2008 (induration in mm).

**Figure 3:**
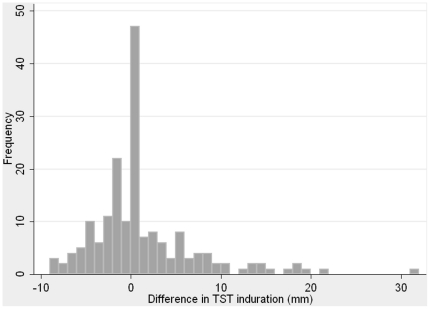
Frequency distribution of change in tuberculin skin test reactions since baseline testing (induration in mm).

Figure 2 shows the frequency distribution of TST indurations at repeat testing in 2008, measured in mm. Ninety-three students (52%) had no reaction to TST, while 34 students (19%) had an induration of 10 mm or higher. Figure 3 shows the frequency distribution for the difference in TST induration between the two test points; the plot shows that there are individuals who have had an increase in induration in the year between testing, however there are also a group of students in the left of the graph with a decrease in induration, whose baseline TST was more than 0 but less than 10 mm, yet upon annual testing their TST induration became smaller or indeed reverted to zero.

Using the standard ATS/CDC/IDSA definition for a TST conversion: a repeat TST induration of 10 mm or higher and an increase in induration of 10 mm over the baseline TST result, we found 14 students met the definition for TST conversion. Therefore using this definition we estimate an annual risk of TB infection (ARTI) of 7.8%, (95% CI: 4.34–12.77). Of the 14 students with conversions, 13 (93%) consented to preventive therapy and completed 4 months of daily Rifampicin therapy. No serious adverse events occurred.

### QFT Results

QFT results were available for 177 out of 179 TST negative nursing students who underwent repeat testing in 2008. Using the manufacturer's cut-off, 131 students (74%) tested negative, 44 tested positive (24.9%) and two had indeterminate responses (1.1%).

Among the 14 students who met the ATS/CDC/IDSA definition for TST conversion, 9 (64.3%) were also positive by QFT in 2008, (range of IFN-gamma: 0.55–14.3 IU/mL). The remaining 5 TST converters, tested negative on QFT-GIT (range: 0.32–0.09 IU/mL).

### Univariate analysis of TB Exposure and TST Conversions

Table 3 presents univariate odds ratios measuring the association between demographic and TB exposure variables and TST conversions (using the ATS/CDC/IDSA conversion definition). No demographic variables were significantly associated with TST conversions in this cohort. Among TB exposure variables: Days spent caring for pulmonary TB patients since baseline testing (OR = 1.10, 95% CI:1.04–1.17) and having performed or assisted in sputum collection since baseline testing (OR = 3.57, 95% CI:1.07–11.84) were both significantly associated with TST conversions.

**Table 3:** Association between risk factors and TST Conversions: Results of univariate logistic regression (Bolded variables reached statistical significance).

Variable	OR	95% CI
Age (yrs)	1.01	(0.81–1.27)
		
BCG vaccination	0.9	(0.1–7.67)
		
BMI >19		
<19	1	-
>19	0.93	(0.78–1.12)
		
Highest level of education completed		
Class 12	1	-
Diploma or higher	1.67	(0.34–8.16)
		
Nursing Course Currently Enrolled		
Diploma	1	-
BSc	0.32	(0.08–1.2)
Post BSc diploma or MSc	0.66	(0.08–5.6)
Average household monthly income (Indian Rupees)		
Low (<5,000)	1	-
Medium (5,000–10,000)	1.26	(0.36–4.4)
High (>10,000)	0.5	(0.09–2.7)
Total time spent in health care (months)	0.999	(0.977–1.02)
Direct contact with sputum positive TB pt	0.93	(0.73–1.19)
**Days spent caring for pulmonary TB pts**	**1.10**	**(1.04–1.17)**
**Ever performed or assisted in sputum collection**	**3.57**	**(1.07–11.84)**
Days spent working on isolation wards	0.998	(0.90–1.11)
Days spent working on pulmonary medicine wards	1.12	(0.91–1.37)
**QFT Positive (using 0.35 IU/ml cut-off)**	**4.15**	**(1.55–11.13)**
**Continuous IFN-gamma result (TB Ag- Nil)**	**1.56**	**(1.2–2.02)**

We also looked at the association between QFT results and TST Conversions in 2008. QFT positivity (using the manufacturer's cut-off, 0.35 IU/ml) showed the strongest association with TST conversions (OR = 4.15, 95% CI:1.55–11.13), while continuous IFN-gamma responses (measured in IU/mL) were also significantly associated with conversions (OR = 1.56, 95% CI:1.2–2.02).

### Multivariate analysis

Table 4 presents the results from the multivariate logistics regression analysis. We included variables that were significant in univariate analyses as well as age and BCG vaccination. Having performed or assisted in sputum collection (OR = 4.57, 95% CI: 1.11–18.86) and QFT positivity (OR = 5.89, 95% CI: 1.72–20.23) were both strongly associated with TST conversions. Days spent caring for pulmonary TB patients was also significantly associated with conversions. Therefore, sputum collection and caring for pulmonary TB patients are both high risk activities that may lead to increased risk for TST conversions in this young nursing cohort.

**Table 4:** Association of TB Exposure and risk factors with TST conversions: Results from multivariate logistic regression (Bolded variables reached statistical significance).

Variable	OR	95% CI
Age (yrs)	1.08	(0.85–1.39)
BCG vaccination	0.45	(0.04–4.62)
**Days spent caring for pulmonary TB pts**	**1.12**	**(1.04–1.20)**
**Ever performed or assisted in sputum collection**	**4.57**	**(1.11–18.86)**
**QFT positivity in 2008 (cut-off: 0.35 IU/ml)**	**5.89**	**(1.72–20.23)**

## Discussion

TB continues to be a major public health concern worldwide. In India, where there are more TB cases than any other country, HCWs are at higher risk for TB exposure compared to the general population. The growing concern worldwide regarding TBIC and nosocomial infection has brought attention to the problem of hospital acquired TB infection among HCWs. While our previous study[15] among Indian nursing trainees showed a high prevalence of LTBI, our current study demonstrated a very high annual risk of TB infection (ARTI) of 7.8%, several times higher than the national average of 1.5%, and represents a significantly elevated occupational risk.

We found TST conversions were associated with the 2 variables capturing occupational TB exposure namely; days spent caring for pulmonary TB patients (OR = 1.12, 95%CI: 1.04–1.20) and having performed or assisted in sputum collection. The latter was strongly associated with TST conversions, as we would expect, given the high risk nature of the procedure (OR = 4.57, 95% CI: 1.11–18.86) and also because nursing trainees at CMC do not routinely use N95 respirators while doing the procedure. This suggests that certain occupational activities may be associated with high risk of TST conversions, even in a high TB incidence setting, and suggests areas for implementation of TBIC.

The longitudinal nature of the study, ascertainment of TB exposure data from detailed clinical log books, maintained prospectively throughout the study and the ability to associate new exposures to new TST conversions represent strengths of our study. Detailed exposure data are particularly difficult to attain in a high incidence settings such as India, and this study is one of the few that have shown that TST conversions are associated with specific occupational exposures.

One study limitation is the TST itself. False positives can happen with the TST, either because of previous BCG vaccination or by sensitization to non tuberculous mycobacteria (NTM). However, BCG vaccination in India is routinely given at birth and not repeated,[21] and should therefore be unlikely to cause false positive TST reactions in adults. Also, NTM is thought to not be clinically important cause of false positives unless the prevalence of NTM is high and the prevalence of TB is low,[22] and this is not the case in Southern India. While an IGRA (QuantiFERON TB Gold) test was included in the study protocol in 2008, we do not have baseline QFT results to compare and therefore could not assess whether TST conversions are associated with QFT conversions. Future analyses of this ongoing cohort will address such questions.

The high ARTI in this cohort indicates ongoing nosocomial transmission at CMC hospital. Health care workers are the backbone in the fight against TB and we must endeavor to protect them while they care for contagious patients. Indian healthcare providers and the Indian Revised National TB Control Programme will need to consider implementing infection control interventions in hospitals and health care centers in order to reduce nosocomial transmission and protect health care workers. With the WHO TBIC policy guidelines now available,[4] the emphasis should be on improving the safety of health care workers in India. Indeed, the Indian RNTCP has recently published guidance on airborne infection control in healthcare settings, but implementation of this guideline has been weak. [23] Our data suggests that the RNTCP guidelines must be urgently implemented, especially among young nursing and healthcare trainees because of their higher risk of TB infection and disease.

A recent modeling study from India examined the likely benefits of IPT as an intervention to reduce TB rates in Indian HCWs.[24] This analysis showed that implementation of IPT after serial tuberculin skin testing, along with other general TBIC measures, can have a large impact, especially in young HCWs and trainees. In this subgroup of young HCWs, the benefits outweighed the potential risks and adverse effects of IPT.

While testing all HCWs in India is daunting, we think a strong case can be made for at least doing annual TSTs on young HCWs and trainees – especially medical and nursing students, interns, allied health sciences students who are at risk of TB exposure, and postgraduates during their residency training.[25] In these groups, TST can be an integral component of pre-training/employment screening at the start of training, and repeated every year to identify those who are newly infected. Such convertors must then be screening to rule out active TB disease, before initiation of preventive therapy.

The rate-limiting step for Indian hospitals and administrators, we suspect, is not lack of resources. After all, TST screening and isoniazid therapy are inexpensive, and most large hospitals already have the required expertise in hospital infection control committees. The real problem may be apathy, unwillingness to act on available evidence, and a fatalistic acceptance that TB risk is part and parcel of being a healthcare professional in India [25].
